# Why Is Water More Reactive Than Hydrogen in Photocatalytic CO_2_ Conversion at Higher Pressures? Elucidation by Means of X-Ray Absorption Fine Structure and Gas Chromatography–Mass Spectrometry

**DOI:** 10.3389/fchem.2018.00408

**Published:** 2018-09-27

**Authors:** Hongwei Zhang, Yasuo Izumi

**Affiliations:** Department of Chemistry, Graduate School of Science, Chiba University, Chiba, Japan

**Keywords:** CO_2_, oxygen vacancy, X-ray absorption fine structure, gas chromatography–mass spectrometry, ^13^CO_2_

## Abstract

Photocatalytic conversion of CO_2_ into mainly methane using Pd/TiO_2_ photocatalyst proceeded faster at 0.80 MPa using water rather than hydrogen as a reductant. The former reaction (CO_2_ + water) consists of two steps: first, water photosplitting and second, the latter reaction (CO_2_ + hydrogen). It was paradoxical that total steps proceeded faster than each step based on simple kinetics. To elucidate the reason, Pd and Ti K-edge X-ray absorption fine structure (XAFS) was monitored during CO_2_ photoconversion using H_2_ or moisture and the exchange reaction of ^13^CO_2_ at Pd/TiO_2_ surface was also monitored. As a result, the coordination number, *N*(Ti–O) and *N*[Ti(–O–)Ti] values, decreased from original values for TiO_2_ crystalline (6 and 12) to 4.9–5.7 and 9.7–10.6 under CO_2_ and moisture, respectively, in contrast to significantly smaller decreases under CO_2_ and H_2_ and under Ar. The exchange of gas-phase ^13^CO_2_ with preadsorbed ^12^CO_2_ reached the equilibrium in ~20 h with a rate constant of 0.20 h^−1^. The reason of the higher activity using water rather than H_2_ could be explained owing to the oxygen vacancy (O_*v*_) sites as confirmed by XAFS. The reaction of TiO_2_ surface with water formed O_*v*_ sites responsible for water oxidation, specially separated from Pd nanoparticle sites for CO_2_ reduction. In contrast, Pd nanoparticle sites were competed by CO_2_ and H species, and the photoconversion of CO_2_ was suppressed at the elevated pressure of CO_2_ + H_2_.

## Introduction

Photocatalytic conversion of CO_2_ into fuels is one of the routes to producing C neutral fuels without a net increase in atmospheric CO_2_ concentrations associated with fossil-derived alternatives (Izumi, 2013). The reaction includes two steps: water oxidation to O_2_ followed by CO_2_ photoconversion to form methane, etc.

(1) $$\begin{array}{ccc} {\text{2H}}_{\text{2}}\text{O(g)} & \to & {\text{O}}_{\text{2}}\text{(g)}+{\text{4H}}^{+}(\text{surface})+4e^{-} \end{array}$$

(2) $$\begin{array}{ccc} {\text{CO}}_{\text{2}}\text{(g)} & + & {\text{8H}}^{+}(\text{surface})+8e^{-}\to {\text{CH}}_{4}(\text{g})+{\text{2H}}_{\text{2}}\text{O(g)} \end{array}$$

We systematically tested the photocatalytic reaction under CO_2_ + H_2_ (Table 1A), an essentially identical reaction to Equation (2), for photocatalytic conversion of CO_2_ into fuels, if it is combined with the water photo-oxidation step (Equation 1) (Kawamura et al., 2017). Based on the formation rates of C-containing products (CH_4_, CO, and methanol) using Pd/TiO_2_, water was evidently more reactive (37 μmol-C h^−1^ g${}_{\text{cat}}^{-1}$) than H_2_ (8.6 μmol-C h^−1^ g${}_{\text{cat}}^{-1}$) for CO_2_ photoconversion at the higher reaction pressures, e.g., 0.80 MPa (Table 1C, G). It was paradoxical that a part of the reaction steps, i.e., Equation (2), was slower than total reaction steps, i.e., Equations (1, 2) based on simple kinetics. In this study, the reason was investigated by X-ray absorption fine structure (XAFS) and gas chromatography–mass spectrometry (GCMS).

**Table 1:** Results of photocatalytic tests of CO_2_ photoconversion for 5 h Using 10 mg of Pd (0.5 weight-%)/TiO_2_ Photocatalysts*^a^ and TiO_2_ (P25) at 0.12–0.80 MPa.

**Entry**	**Catalyst**	**Reactants**			**Formation rates (**μ**mol h**^**−1**^** g**${}_{\text{cat}}^{-1}$**)**				**References**
		**(MPa)**		**(kPa)**	**CO**	**CH_3_OH**	**CH_4_**	Σ(C-products)^*b^	
		**CO_2_**	**H_2_**	**H_2_O**					
A	Pd/TiO_2_	0.12	0.28		<0.08	<0.004	38	38	Kawamura et al., 2017
a′					<0.08	0.034	5.2	5.3	This work
B	TiO_2_				<0.08	0.027	4.0	4.0	Kawamura et al., 2017
C	Pd/TiO_2_	0.24	0.56		<0.08	<0.004	8.6	8.6	Kawamura et al., 2017
D		0.12		2.3	<0.08	0.065	14	14	Zhang et al., 2017
E		0.40			4.1	0.063	19	23	Zhang et al., 2017
F	TiO_2_				<0.08	<0.004	<0.12	<0.20	Zhang et al., 2017
G	Pd/TiO_2_	0.80			6.3	0.52	30	37	Zhang et al., 2017

*a *Prepared using P25 TiO_2_ except for entry a′ (homemade TiO_2_)*.

*b *Total formation rates for C-containing products*.

## Material and methods

A sample was prepared via liquid phase reduction (Kawamura et al., 2017) from 1.0 mM aqueous solution of Na_2_PdCl_4_ (>98%, Sigma Aldrich) and TiO_2_ (P25, Degussa; anatase: rutile phase = 4:1, specific surface area 60 m^2^ g^−1^) stirred at 290 K for 24 h. Then, 40 mM of NaBH_4_ aqueous solution was added in a molar ratio of Pd: NaBH_4_ = 1:8, and the suspension was washed with deionized water (<0.055 μS cm^−1^) supplied by an RFU424TA system (Advantec) before drying under vacuum at 290 K for 1 h and then filtered. The obtained precipitate was washed with deionized water before drying under vacuum at 290 K for 24 h. The obtained sample was denoted as Pd/TiO_2_ and the Pd content was 0.5 weight-% as metal.

For comparison, TiO_2_ was also synthesized in the laboratory. Acid solution (1.2 mL of 5 weight-% H_2_SO_4_ aqueous solution) and 1.37 mL of TiCl_4_ were added into 96 mL of deionized water at 273 K under argon atmosphere and stirred at 900 rotations per minute for 30 min. Then, 28 weight-% ammonia aqueous solution was dropped into the solution at 368 K to adjust the pH 7.0. The solution was stirred at the temperature for 1 h and cooled to 290 K. The obtained suspension was filtered and washed by deionized water (total 500 mL) until no chlorine ions were detected using silver nitrate. The thus-obtained powder was dried at 373 K overnight, calcined at 573 K for 2 h, and white TiO_2_ powder was obtained. The Pd was loaded on the homemade TiO_2_ in a similar manner to the liquid phase reduction for P25 as described earlier.

The high-pressure photoconversion tests were performed in homemade stainless steel reactor equipped with quartz windows (Kawamura et al., 2017; Zhang et al., 2017). A mixed CO_2_/H_2_ gas or CO_2_ gas/moisture was introduced into the reactor. A film (10 mg of photocatalyst) in the reactor was irradiated by a 500-W xenon arc lamp (Model OPM2-502, Ushio) for 5 h. The reaction gas was analyzed using packed columns of 13X-S molecular sieves and polyethylene glycol (PEG-6000) supported on Flusin P (GL Sciences, Inc.) set in a gas chromatograph equipped with a thermal conductivity detector (GC-TCD; Model GC-8A, Shimadzu).

The Pd K-edge XAFS spectra were measured at 290 K in the transmission mode in the Photon Factory Advanced Ring at the High Energy Accelerator Research Organization (KEK) on beamline NW10A. An Si (3 1 1) double-crystal monochromator and a Pt-coated focusing cylindrical mirror were inserted into the X-ray beam path. A Piezo transducer was used to detune the X-ray intensity to two-thirds of the maximum to suppress higher harmonics (Izumi et al., 2001; Fujishima et al., 2015). The Pd K-edge absorption energy was calibrated to 24348 eV using the spectrum of a Pd metal foil (Bearden, 1967).

The Ti K-edge XAFS spectra were measured in the transmission mode in the Photon Factory at KEK on beamline 9A, 9C, and 12C. An Si(1 1 1) double-crystal monochromator and a pair of bent conical/cylinder mirrors and/or double flat mirrors were inserted into the X-ray beam path. A Piezo transducer was used to detune to two-thirds of the maximum X-ray intensity to suppress higher harmonics (Izumi et al., 2001; Fujishima et al., 2015). Spectra for the Pd/TiO_2_ sample (10 mg) diluted by boron nitride were measured under CO_2_ (100 kPa) and moisture (2.2 kPa), under CO_2_ (70 kPa) and H_2_ (30 kPa), or Ar (100 kPa) in the presence/absence of UV–visible light irradiation provided by a 500-W Xe arc lamp (the flux intensity 81.6 mW cm^−2^) at the beamline (Izumi et al., 2007; Morikawa et al., 2014). The Ti K-edge absorption energy was calibrated to 4964.5 eV using the spectrum of a Ti metal foil (Bearden, 1967). The obtained Pd and Ti K-edge XAFS data were analyzed using XDAP software package using modified Victoreen function, spline function, Fourier transform, and multiple-shell curve fitting (Vaarkamp et al., 2006; Izumi et al., 2009).

Isotope-labeled exchange reaction tests were performed and monitored with GCMS using a JMS-Q1050GC (JEOL) apparatus equipped with a quadruple mass spectrometer. Helium (purity > 99.9999%) was used as the carrier gas. The sampling loop comprised a Pyrex glass system vacuumed using rotary and diffusion pumps (10^−6^ Pa) and connected to the JMS-Q1050GC using 1.5 m of deactivated fused silica tube (No. 160-2845-10, Agilent; internal diameter 250 μm) maintained at 393 K during the analysis to avoid gas adsorption (Wein et al., 2018).

A photocatalyst sample (0.100 g) was placed in a quartz photoreactor and evacuated at 295 K for 2 h while being connected to a Pyrex glass circulating system (circulating section 206.1 mL) and rotary and diffusion pumps (10^−6^ Pa). Then, ^13^CO_2_ (0.68 kPa; ^13^C 99%, ^17^O 0.1%, ^18^O 0.7%, chemical purity > 99.9%, Cambridge Isotope Laboratories, Inc.) photoexchange with the photocatalyst surface was monitored at 295 K under irradiation by UV–visible light from a 500 W xenon arc lamp (Model SX-UID502XAM, Ushio). The distance between the exit of the lamp window and the photocatalyst was 18 mm. The exchange with adsorbed ^12^CO_2_ at the surface was monitored using a packed column of PEG-6000 supported on a Flusin P (GL Sciences Inc.) set in the JSM-Q1050GC.

The exchange reaction rates were assumed to follow first-order equilibrium kinetics and the rate constants *k*_*r*_ and *k*_*r*_′ are for the exchange of gas-phase ^13^CO_2_ with adsorbed ^12^CO_2_ and gas-phase ^12^CO_2_ with adsorbed ^13^CO_2_, respectively.

(3) $$\begin{array}{ccc} \frac{dP_{{}^{13}{\text{CO}}_{2}}}{dt} & = & -k_{r}P_{{}^{13}{\text{CO}}_{2}}+k_{r}{}^{\prime }P_{{}^{12}{\text{CO}}_{2}} \end{array}$$

(4) $$\begin{array}{ccc} P_{{}^{12}{\text{CO}}_{2}} & = & \frac{k_{r}}{k_{r}+k_{r}{}^{\prime }}\left\{1-e^{-(k_{r}+k_{r}{}^{\prime })t}\right\}P_{{}^{13}{\text{CO}}_{2}(\text{initial)}} \end{array}$$

(5) $$\begin{array}{ccc} P_{{}^{12}{\text{CO}}_{2}(\text{eqilibrium})} & = & \frac{k_{r}}{k_{r}+k_{r}{}^{\prime }}P_{{}^{13}{\text{CO}}_{2}(\text{initial})} \end{array}$$

(6) $$\begin{array}{ccc} ∴P_{{}^{12}{\text{CO}}_{2}} & = & P_{{}^{12}{\text{CO}}_{2}(\text{eqilibrium})}\left\{1-e^{-(k_{r}+k_{r}{}^{\prime })t}\right\} \end{array}$$

Photocatalytic tests using ^13^CO_2_ (2.3 kPa) and H_2_ (21.7 kPa) were also performed in a similar apparatus and conditions to that of ^13^CO_2_ exchange tests.

## Results and discussion

### Photoconversion tests of CO_2_

First, reported kinetic results are summarized. Both the reactions under CO_2_ + H_2_ and under CO_2_ + moisture critically depended on the pressure of reactants using Pd/TiO_2_ (Kawamura et al., 2017; Zhang et al., 2017). The formation rates of C-containing products under CO_2_ + H_2_ reached a local maximum at 0.40 MPa (0.12 MPa of CO_2_, 0.28 MPa of H_2_) and drew a volcanolike dependence (Table 1A, C). In Kawamura et al. (2017), the volcanolike local maximum seems distributed relatively steep based on 13 test data points. Methane was a dominant product under the conditions. In contrast, the formation rates followed a typical Langmuir-type dependence, i.e., they gradually increased as a function of reactants pressure under CO_2_ + moisture at 0.12–0.80 MPa (Table 1D, E, G) (Zhang et al., 2017). Minor CO and methanol were also produced under the conditions.

Due to the balance of different pressure dependencies between the two types of reactions, H_2_ was more reactive at lower than 0.40 MPa, whereas water was more reactive at higher than 0.40 MPa as a reductant for CO_2_. In this context, the major question of this study is the reason for the different pressure dependencies of CO_2_ photoconversion rates using H_2_ vs. water.

Photoconversion test was performed at 0.40 MPa of CO_2_ and H_2_ using Pd/TiO_2_ with the help of homemade TiO_2_ (Table 1a′). Methane was the major product similar to the result using Pd/TiO_2_ prepared from commercial P25 (Table 1A) (Kawamura et al., 2017). The reason for the difference of the methane formation rates (5.2 μmol h^−1^ g${}_{\text{cat}}^{-1}$ vs. 38 μmol h^−1^ g${}_{\text{cat}}^{-1}$) is not clear, but the crystalline phase (anatase vs. the mixture of anatase and rutile phases) and specific surface area were different. Although the impurity effects for photocatalysts using P25 were suggested (Izumi, 2013; Cybula et al., 2015; Li et al., 2016; Dilla et al., 2017; Grigioni et al., 2017) photoconversion of CO_2_ into methane was confirmed using Pd/TiO_2_ prepared by not making use of any organic reagents throughout the synthesis procedure of the photocatalyst (Table 1a′). For this problem, several black tests were reported using Pd/TiO_2_ (P25), especially the one under UV–visible light and H_2_ excluding CO_2_ gas (Zhang et al., 2017). No C-containing products were detected above the detection limit of GC-TCD. Thus, a direct impurity effect is denied as the reason for the rate difference (Table 1A, a′). The indirect effect of gas-phase CO_2_ on impurity on/in catalyst, leading to the formation of C-containing products under light cannot be denied as the reason for formation rate difference.

The other possibility of formation rate difference is critical pressure dependence of TiO_2_-based photocatalysts under CO_2_ + H_2_ (Kawamura et al., 2017). Due to the critical difference of reactant pressure for the conversion rate maximum (volcano top) for each catalyst, formation rate difference of by a factor of 7.2 times between Pd/TiO_2_ (P25) and Pd/TiO_2_ (homemade) may result at a fixed reactant pressure (0.40 MPa).

### Active site monitoring by XAFS

For the morphological characterization of the photocatalysts, transmission electron microscope images for the Pd/TiO_2_ catalyst, fresh and used one, after photocatalytic test in CO_2_ + H_2_ and cross-sectional scanning electron microscope (SEM) images for fresh Pd/TiO_2_ were reported in Kawamura et al. (2017). The morphology of the catalyst negligibly changed after the photocatalytic test, but the mean Pd particle size slightly increased starting from 3.1 to 3.9 nm after the photocatalytic test (Kawamura et al., 2017). Cross-sectional SEM images for fresh Pd/TiO_2_ and used one after photocatalytic test in CO_2_ + moisture were also reported in Zhang et al. (2017). The color of the top few micrometers of Pd/TiO_2_ catalyst film (total 11–16 μm) changed after the photocatalytic test (Zhang et al., 2017). In this study, monitoring of local active sites was performed in addition to these morphological studies.

The X-ray absorption near-edge structure (XANES) at the Pd K-edge suggested metallic Pd nanoparticles dispersed on TiO_2_. The extended X-ray absorption fine structure (EXAFS) also did not show significant change during photoreactions under CO_2_ + H_2_ or CO_2_ + moisture based on the Pd–Pd shell, but the coordination number *N*(Pd–O) decreased from 1.9 for fresh sample to 0.6–0.9 after the photocatalytic tests under CO_2_ + H_2_ at 0.024–0.40 MPa (Kawamura et al., 2017) and to 0.8–1.0 under CO_2_ + moisture at 4.6 kPa−0.40 MPa (Zhang et al., 2017). The decrease of *N*(Pd–O) values suggested the formation of oxygen vacancy (O_*v*_) sites on/in TiO_2_ near Pd nanoparticles under the reaction conditions of CO_2_ photoconversion.

**Figure 1:**
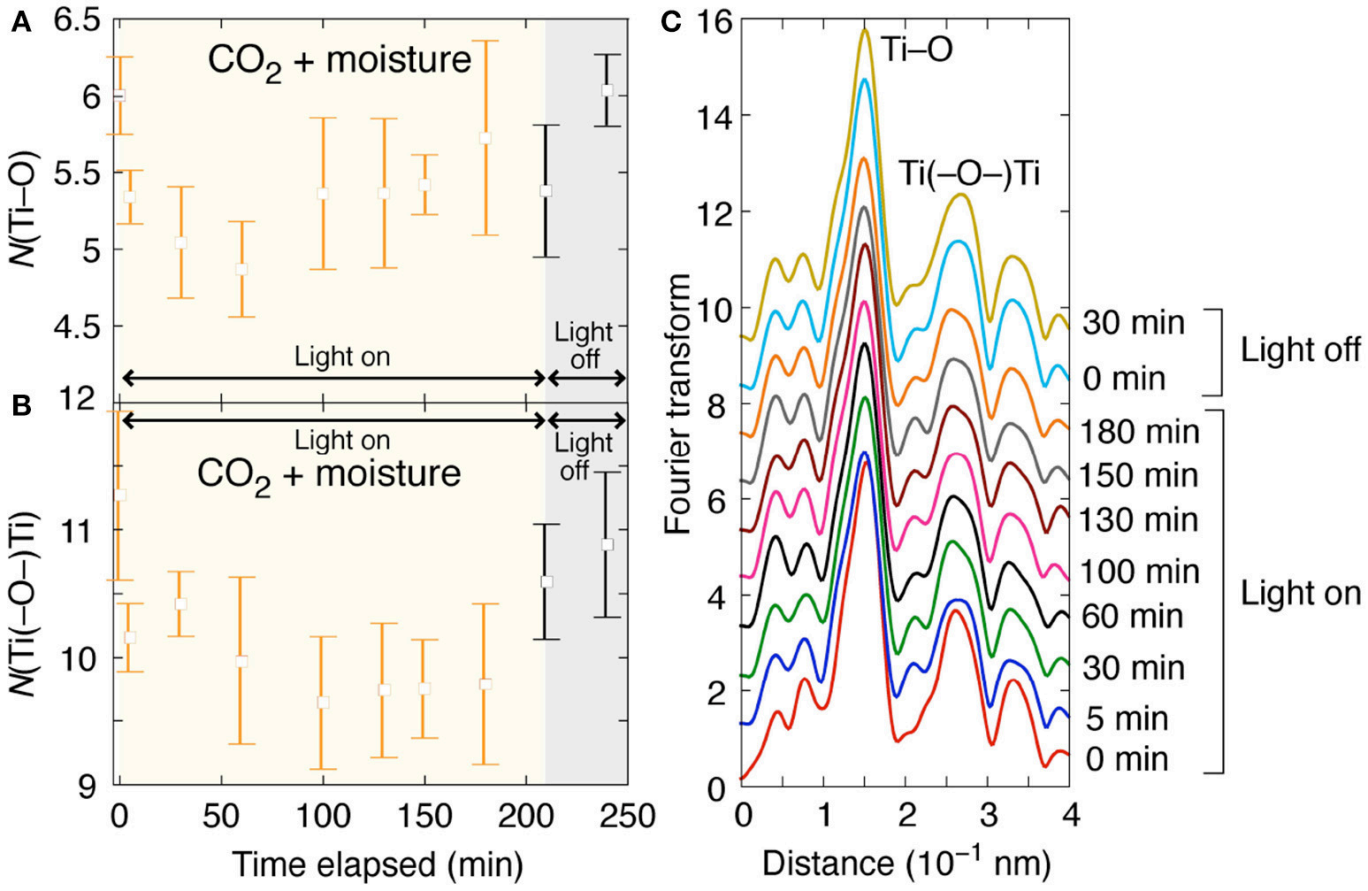
Time course of Ti K-edge EXAFS for Pd/TiO_2_ photocatalyst (10 mg) under CO_2_ (100 kPa) and moisture (2.2 kPa) for 3.5 h irradiated by UV–visible light and subsequently for 0.5 h under dark. The changes of **(A) ***N*(Ti–O) values, **(B) ***N*[Ti(–O–)Ti] values, and **(C)** Fourier transform of angular wavenumber *k*^3^-weighted EXAFS χ function (Zhang et al., 2017).

The Ti K-edge XAFS was also investigated under the reaction conditions of CO_2_ photoconversion with moisture (Figure 1). Under CO_2_ (100 kPa), moisture (2.2 kPa), and UV–visible light, the change in *N*(Ti–O) values was monitored (Figure 1A). The changes under light and then under dark were depicted as the Fourier transform in Figure 1C. The intensity of peak for Ti–O interatomic pair at 0.15 nm (phase shift uncorrected) decreased when the light was on (Figure 1C, 5 min), remained almost constant during the light irradiation, and increased to the original when the light was off (Figure 1C, Light off, 0 min). In accordance with the peak intensity change, in comparison with the initial *N*(Ti–O) value (6.0) under CO_2_, moisture, and dark, the value significantly decreased to 4.9–5.7 during light irradiation for 3.5 h. At 0.5 h after the light was off, the *N*(Ti–O) value increased to the original value (6.0).

The peak intensity of Ti(–O–)Ti interatomic pair at 0.26 nm (phase shift uncorrected; Figure 1C) and the *N*[Ti(–O–)Ti] values followed a similar trend: decrease from the initial 11.3 to 9.7–10.6 during the irradiation of UV–visible light (Figure 1B). Taking the experimental and fit errors into account (Figures 1A,B), the *N*(Ti–O) and *N*[Ti(–O–)Ti] values would evaluate the concentration of O_*v*_ sites in/on TiO_2_ (Zhang et al., 2017).

**Figure 2:**
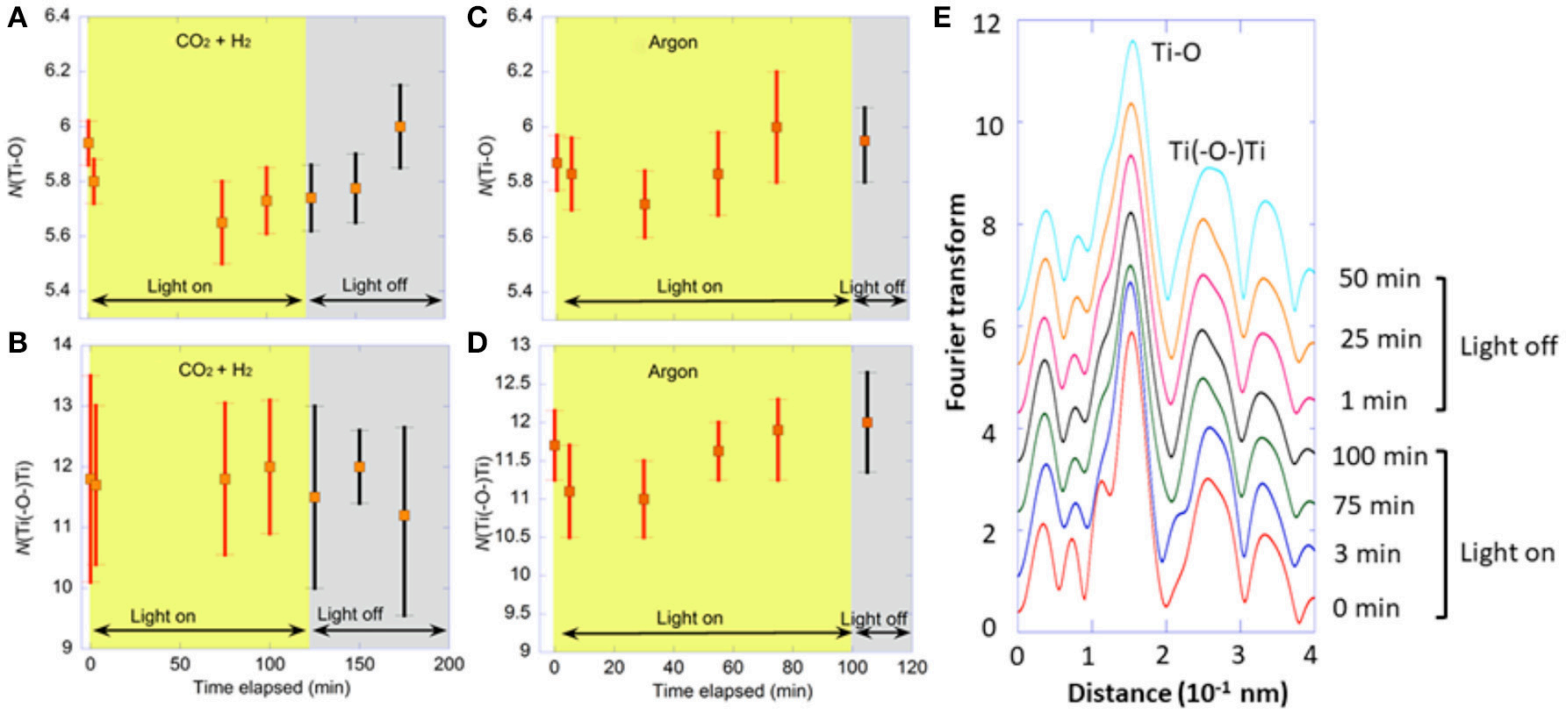
Time course of Ti K-edge **(A–D)** EXAFS for Pd/TiO_2_ photocatalyst (10 mg). **(A,B)** Under CO_2_ (70 kPa) and H_2_ (30 kPa) for 125 min irradiated by UV–visible light and subsequently for 75 min under dark. **(C,D)** Under Ar (100 kPa) for 100 min irradiated by UV–visible light and subsequently for 20 min under dark. The changes of **(A,C) ***N*(Ti–O) values and **(B,D) ***N*[Ti(–O–)Ti] values. **(E)** Fourier transform of angular wavenumber *k*^3^-weighted EXAFS χ function under CO_2_ (70 kPa) and H_2_ (30 kPa).

Two control monitoring tests were also performed: (i) using H_2_ instead of moisture as reductant for CO_2_ and (ii) using only argon. First, under CO_2_ (70 kPa), H_2_ (30 kPa), and UV–visible light, the changes in peak intensity of Ti–O and Ti(–O–)Ti interatomic pair at 0.15 nm and 0.26 nm (phase shift uncorrected), respectively (Figure 2E) and the resultant *N*(Ti–O) and *N*[Ti(–O–)Ti] values were monitored (Figures 2A,B). The peak intensity of Ti–O interatomic pair decreased at 75 min of light irradiation, but no clear correlation between light irradiation and the Ti–O peak intensity was found. The peak intensity of Ti(–O–)Ti interatomic pair did not change much. In accordance with the peak intensity changes, in comparison with the initial value (5.9) under CO_2_, H_2_, and dark, the *N*(Ti–O) value varied, but within a small range of 5.6–5.8 during light irradiation for 125 min. At 50 min after the light was off, the *N*(Ti–O) value increased to the original value (6.0). In comparison with the monitoring under CO_2_ and moisture (starting from 6.0 to 4.9–5.7; Figure 1A), the decrease in *N*(Ti–O) value was effectively smaller under CO_2_ and H_2_ (Δ = 0.1–0.3) during photoirradiation. The *N*[Ti(–O–)Ti] value remained between 12 and 11.7 under the irradiation of UV–visible light and negligibly changed after the light was off: 12–11.2 (Figure 2B). Under CO_2_ and moisture, the decrease was greater: from 11.3 to 9.7–10.6 by the effect of light (Figure 1B).

Under Ar (100 kPa) and UV–visible light, the *N*(Ti–O) value changed between 5.7 and 6.0 during light irradiation for 100 min. At 5 min after the light was off, the *N*(Ti–O) value remained at 5.9 (Figure 2C). The changes in *N*[Ti(–O–)Ti] values were also minimal under Ar: 12–11 throughout the monitoring test under light/dark (Figure 2D). These differences in *N*(Ti–O) and *N*[Ti(–O–)Ti] values were because more O_*v*_ sites were formed under the photoreduction of CO_2_ using moisture rather than H_2_ or Ar.

### Monitoring of ^13^CO_2_ exchange by GCMS

**Figure 3:**
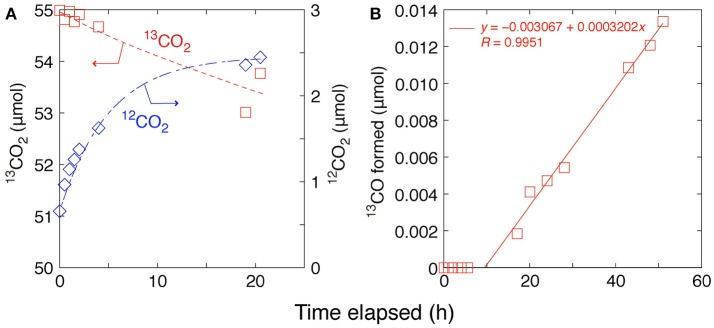
Time courses of **(A)** ^13^CO_2_ (0.68 kPa) photoexchange reaction with preadsorbed ^12^CO_2_ on Pd/TiO_2_ (P25) and **(B)** photocatalytic reduction test in ^13^CO_2_ (2.3 kPa) and H_2_ (21.7 kPa) on Pd/TiO_2_ (homemade).

The ^13^CO_2_ exchange reaction test was performed using Pd–TiO_2_ (P25) photocatalyst. The amount of ^13^CO_2_ gas gradually decreased over 20 h by the exchange with ^12^CO_2_ on Pd–TiO_2_ sample that was preadsorbed from air (Figure 3A). The changes followed equilibrium kinetics as listed in Equations (3–6), and the formation of ^12^CO_2_ (*n*: mol) in gas phase was well fitted by Equation (6′) (Figure 3A).

(6′) $$n_{{}^{12}CO_{2}}(\mu mol)=0.657+1.80(1-e^{-0.20t})$$

Initial 0.657 μmol is the impurity (1.18 mol%) included in the introduced ^13^CO_2_ gas (0.68 kPa). The $n_{{}^{12}CO_{2}\left(equiliburium\right)}$ value (1.80 μmol) obtained by the fit of Equation (6′) to the data corresponds to 0.18 molecules of CO_2_ adsorbed per nm^2^ of Pd–TiO_2_ that was an acceptable value after the evacuation at 295 K for 2 h as pretreatment. The sum of rate constants *k*_*r*_ and *k*_*r*_′ corresponds to total rate constant (Equation 6) to reach the equilibrium ^13^CO_2_ and ^12^CO_2_ (0.20 h^−1^).

As a comparison, if we assume that the dispersion of mean 3.1 nm Pd nanoparticles (0.50 weight-%) over TiO_2_ observed by transmission electron microscopy (Kawamura et al., 2017) is ~50% (Kip et al., 1987) and the conversion to C-containing products (methane, CO, and methanol) proceeded on the surface of the Pd atoms starting from CO_2_, the formation rate of C-containing products (37 μmol h^−1^ g${}_{\text{cat}}^{-1}$) under 0.80 MPa of CO_2_ and moisture (Table 1G) corresponds to the turnover frequency (TOF)

$\frac{37\times 10^{-6}mol\;h^{-1}{g_{cat}}^{{}^{-1}}\times 10^{-2}g_{cat}}{\frac{10^{-2}g_{cat}\times 0.005\times 0.5}{106.42{\text{g}}_{Pd}\text{mo}{\text{l}}^{-1}}}$ = 1.6 h^−1^. This comparison is contradictory because the early adsorption equilibrium of CO_2_ (0.20 h^−1^ based on Equation 6′) is slower than CO_2_ photoreduction to methane via much more difficult reaction steps (1.6 h^−1^). However, the exchange rate (0.20 h^−1^) observed under ^13^CO_2_ (initial 0.68 kPa) in this study was by a couple of times higher compared with the formation rate of C-containing products under initial 3.4 kPa of CO_2_ + 1.2 kPa of moisture (0.9 μmol h^−1^ g${}_{\text{cat}}^{-1}$) (Zhang et al., 2017) corresponding to the TOF

$\frac{0.9\times 10^{-6}mol\;h^{-1}{g_{cat}}^{{}^{-1}}\times 10^{-2}g_{cat}}{\frac{10^{-2}g_{cat}\times 0.005\times 0.5}{106.42{\text{g}}_{Pd}\text{mo}{\text{l}}^{-1}}}$ = 0.04 h^−1^. The CO_2_ exchange was reported using isotope labeled Ti^18^O_2_ irradiated by XeCl excimer laser (308 nm) to form C^18^O_2_ and ^16^OC^18^O (Civiš et al., 2011).

Furthermore, stability test of photocatalytic reaction using ^13^CO_2_ (2.3 kPa) + H_2_ (21.7 kPa) and Pd/TiO_2_ was performed for more than 50 h (Figure 3B). To avoid the possibility of impurity effects, Pd/TiO_2_ (homemade) was used and a byproduct ^13^CO was monitored by GCMS rather than methane that may be produced from impurity in TiO_2_ (Izumi, 2013; Cybula et al., 2015; Li et al., 2016; Dilla et al., 2017; Grigioni et al., 2017). In accordance with the rate of ^13^CO_2_ exchange reaction (Figure 3A), ^13^CO started to be formed at ~10 h of reaction and constantly formed for more than 50 h (Figure 3B). As a byproduct, the formation rate was low 0.0032 μmol h^−1^ g${}_{\text{cat}}^{-1}$ corresponding to the TOF 0.00014 h^−1^. Nevertheless, the stability test confirmed the availability of Pd/TiO_2_ photocatalyst for more than 50 h of photoreaction test.

Thus, we should be careful in the isotope tracing tests as ^12^CO_2_ could be included derived from preadsorbed ^12^CO_2_ in the reactant ^13^CO_2_ and ^12^C-products could be included by the photocatalytic conversion.

The Pd/TiO_2_ catalyst used in this study is advantageous for CO_2_ photoconversion irradiated by visible light (Kawamura et al., 2017; Zhang et al., 2017). The photocatalytic performance was compared with TiO_2_ (P25) in 0.40 MPa of CO_2_ + H_2_ (Table 1A, B) or CO_2_ + moisture (Table 1E, F). The formation rates of total C-containing products were 11% and <0.9% using TiO_2_ in comparison with corresponding values using Pd/TiO_2_, respectively. As TiO_2_ is mostly activated by UV light based on the band gap value (3.2 and 3.0 eV for anatase and rutile phases, respectively) (Izumi, 2013), the increased photoactivity using Pd/TiO_2_ could be the effects of localized surface plasmon resonance of Pd nanoparticles owing to visible light irradiation (Kawamura et al., 2017; Zhang et al., 2017).

Thus, the answer to the question as to why water was more reactive than H_2_ in photocatalytic CO_2_ conversion at higher pressures is that water forms hydroxy radical under the irradiation of UV–visible light (Equation 7) and then O_*v*_ sites are formed (Equation 8), but not by using H_2_ or Ar. The exchange of CO_2_ (Figure 3A) and/or the elimination of C–O bond to form CO are also suggested over O_*v*_ sites of Au–Cu–TiO_2_ (Neatu et al., 2014), MgO–Pt–TiO_2_ (Xie et al., 2014), TiO_2_ (Liu et al., 2012, 2016; Li et al., 2017), and theoretical models of defective anatase (Ji and Luo, 2016), anatase (1 0 1), and small TiO_2_ cluster (Lee and Kanai, 2012).

(7) $$\begin{array}{ccc} {\text{H}}_{\text{2}}\text{O} & + & h^{+}\to {\text{H}}^{+}+\cdot \text{OH} \end{array}$$

(8) $$\begin{array}{ccc} \cdot \text{OH} & + & \text{O}(\text{surface})+3h^{+}\to {\text{O}}_{\text{2}}\text{(g)}+{\text{O}}_{\text{v}}+{\text{H}}^{\text{+}}\text{(surface)} \end{array}$$

The reactions 7 and 8 proceeded as water oxidation over TiO_2_ spatially separated from CO_2_ reduction sites on Pd for Equation (2). Thus, the effective redox reaction of Equations (1, 2) proceeded using water. Both the reactions 1 and 2 would proceed competitively on Pd under CO_2_ + H_2_, and the efficiency followed a volcano-like dependence as a function of partial pressures of reactants (Table 1A, B) (Kawamura et al., 2017).

## Conclusions

The formation of O_*v*_ sites was monitored by means of Ti K-edge EXAFS using Pd/TiO_2_. The coordination number around Ti atoms clearly demonstrated the effective formation of O_*v*_ sites under CO_2_, moisture, and UV–visible light in contrast to the much smaller population of O_*v*_ sites under CO_2_, H_2_, and UV–visible light. This difference explained the effective red-ox site separation for water oxidation via ·OH radicals and O_*v*_ sites and CO_2_ reduction over Pd nanoparticle sites under CO_2_, moisture, and UV–visible light. The exchange of ^13^CO_2_ with preadsorbed ^12^CO_2_ over Pd/TiO_2_ reached the equilibrium in ~20 h. This result suggested the earlier step of CO_2_ photoconversion, but we need to be aware that ^12^C-products are formed via photocatalytic reaction tests under ^13^CO_2_.

## Author contributions

This study was planned by YI, most of the experiments and analyses were performed by HZ, and this manuscript was written together.

### Conflict of interest statement

The authors declare that the research was conducted in the absence of any commercial or financial relationships that could be construed as a potential conflict of interest.
